# Combined effects of spray‐drying conditions and postdrying storage time and temperature on *Salmonella choleraesuis* and *Salmonella typhimurium* survival when inoculated in liquid porcine plasma

**DOI:** 10.1111/lam.13017

**Published:** 2018-06-27

**Authors:** E. Blázquez, C. Rodríguez, J. Ródenas, N. Saborido, M. Solà‐Ginés, A. Pérez de Rozas, J. M. Campbell, J. Segalés, J. Pujols, J. Polo

**Affiliations:** ^1^ APC EUROPE Granollers Barcelona Spain; ^2^ IRTA Centre de Recerca en Sanitat Animal (CReSA‐IRTA) Campus de la Universitat Autònoma de Barcelona Bellaterra Barcelona Spain; ^3^ APC Inc. Ankeny IA USA; ^4^ Departament de Sanitat i Anatomia Animals Universitat Autònoma de Barcelona (UAB) Bellaterra Barcelona Spain; ^5^ UAB Centre de Recerca en Sanitat Animal (CReSA, IRTA‐UAB) Campus de la Universitat Autònoma de Barcelona Bellaterra Barcelona Spain

**Keywords:** blood derivative, *Salmonella choleraesuis*, *Salmonella typhimurium*, spray‐drying, storage conditions

## Abstract

The objective of this study was to determine the effectiveness of the spray‐drying process on the inactivation of *Salmonella choleraesuis* and *Salmonella typhimurium* spiked in liquid porcine plasma and to test the additive effect of immediate postdrying storage. Commercial spray‐dried porcine plasma was sterilized by irradiation and then reconstituted (1:9) with sterile water. Aliquots of reconstituted plasma were inoculated with either *S. choleraesuis* or *S. typhimurium,* subjected to spray‐drying at an inlet temperature of 200°C and an outlet temperature of either 71 or 80°C, and each spray‐drying temperature combinations were subjected to either 0, 30 or 60 s of residence time (RT) as a simulation of residence time typical of commercial dryers. Spray‐dried samples were stored at either 4·0 ± 3·0°C or 23·0 ± 0·3°C for 15 days. Bacterial counts of each *Salmonella* spp., were completed for all samples. For both *Salmonella* spp., spray‐drying at both outlet temperatures reduced bacterial counts about 3 logs at RT 0 s, while there was about a 5·5 log reduction at RT 60 s. Storage of all dried samples at either 4·0 ± 3·0°C or 23·0 ± 0·3°C for 15 days eliminate all detectable bacterial counts of both *Salmonella* spp.

**Significance and Impact of the Study:**

Safety of raw materials from animal origin like spray‐dried porcine plasma (SDPP) may be a concern for the swine industry. Spray‐drying process and postdrying storage are good inactivation steps to reduce the bacterial load of *Salmonella choleraesuis* and *Salmonella typhimurium*. For both *Salmonella* spp., spray‐drying at 71°C or 80°C outlet temperatures reduced bacterial counts about 3 log at residence time (RT) 0 s, while there was about a 5.5 log reduction at RT 60 s. Storage of all dried samples at either 4.0 ± 3.0°C or 23.0 ± 0.3°C for 15 days was effective for eliminating detectable bacterial counts of both *Salmonella* spp.

## Graphical abstract

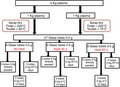

## Introduction

Spray‐dried blood products (SDBP) are used in human food and animal feed. Ingredients like spray‐dried plasma (SDP) or spray‐dried red blood cells are used in the food and meat industry to provide texture, emulsion capacity and natural colour properties (Appiah and Peggy 2012). Likewise, SDP is an ingredient extensively used globally in pig feed due to its well‐known beneficial effects on postweaning performance and survival (Torrallardona 2010). In contrast, pathogen contamination of animal‐based ingredients is a major safety concern for both food and feed industries. During the last decade, a significant amount of data has been published about the safety of commercial spray‐dried blood products relative to bacteria (Polo *et al*. 2002), and enveloped (Polo *et al*. 2005; Gerber *et al*. 2014; Opriessnig *et al*. 2014; Pujols and Segalés 2014) and non‐enveloped viruses (Pujols *et al*. 2008, 2011, 2014; Shen *et al*. 2011; Pérez‐Bosque *et al*. 2016) affecting the swine industry. These studies have demonstrated the importance of several features of the manufacturing process of commercial blood products that contribute to the bio‐safety of these functional ingredients. In addition, the liquid blood of multiple pigs slaughtered per day is pooled. Pooling of blood that inherently contains antibodies with neutralizing capacity against a variety of pathogens contributes to the biosafety of the finished product (Williams and Khan 2010; Polo *et al*. 2013).

Spray‐drying is based on the desiccation of a solution or suspension into a dried particulate form by spraying the feed into a hot drying chamber. The spray‐drying process involves four stages of operation as follows: (i) atomization of liquid source into a hot chamber; (ii) contact between the spray and the drying medium (very hot air at a high gas mass to liquid mass flow volume ratio); (iii) moisture evaporation resulting in particle formation; and (4) separation of dried products from the air stream (Kuriakose and Anandharamakrishnan 2010). During the spray‐drying process, computer systems designed to control and monitor processing temperatures and conditions are used to ensure that SDBP have been exposed to a minimum of 80°C throughout its substance. This is one of the most important critical control points in the manufacturing process of SDBP intended for human or animal consumption.

Also, SDBP have low moisture (<9%) and very low water activity (*a*_w_ < 0·6). Some pathogens, especially bacteria and enveloped viruses, are not able to survive for a prolonged time in dried materials like SDBP (Perdana *et al*. 2013; Sampedro *et al*. 2015; Pérez‐Bosque *et al*. 2016). Therefore, most SDBP manufacturers have adopted postprocessing storage of SDP of porcine origin at room temperature (>20°C) for at least 2 weeks after production as an additional safety feature (Sampedro *et al*. 2015). Thus, the sequential action of spray‐drying and storage at room temperature for at least 2 weeks after spray‐drying and packaging is able to inactivate micro‐organisms.

*Salmonella enterica subsp. enterica Serovar Typhimurium* (*S. typhimurium*) is a cause of acute food‐borne zoonosis worldwide (Hohmann 2001) and pigs are important reservoirs (Gebreyes *et al*. 2004). *S. typhimurium* is the second most common serotype associated with food‐borne illness. *Salmonella enterica subsp. enterica Serovar Choleraesuis* (*S. choleraesuis*) is frequently reported in North America and Asia (Gray *et al*. 1995; Boyen *et al*. 2008) as causing disease in pigs, with a lower prevalence reported in Europe. *S*. *choleraesuis* has also been reported to cause systemic infections in humans (Chiu *et al*. 2004).

The objective of this study was to determine the effectiveness of the spray‐drying process on the inactivation of *S. choleraesuis* and *S. typhimurium* spiked in liquid porcine plasma. In addition, a second objective was to test the additive effect of immediate postdrying storage of the dried samples at two different storage temperatures 4·0 ± 3·0°C or 23·0 ± 0·3°C (room temperature) for 15 days on the inactivation of both *Salmonella* strains.

## Results and discussion

All samples before *Salmonella* spp. inoculation showed an initial total plate count <10 CFU ml^−1^, which was the limit of detection.

Plasma inoculated with *S. choleraesuis* strain had an initial count of 10·12 ± 0·17 log_10_ ml^−1^ and the average count of plasma inoculated with *S. typhimurium* was 9·56 ± 0·17 log_10_ ml^−1^ (Table 1).

**Table 1:** Effect of spray‐drying porcine plasma at 200 ± 5°C inlet temperature and two different outlet temperatures (80** ± **1 and 71** ± **1°C), and kept at 3 different residence times (0, 30 or 60 s) on the inactivation of *Salmonella choleraesuis* and *Salmonella typhimurium*

	*S. choleraesuis* CFU Log_10_/g solids	RF	*S. typhimurium* CFU Log_10_/g solids	RF
Inoculated plasma	10·12 ± 0·17		9·56 ± 0·17	
71°C SDPP at 0 s RT	7·90 ± 0·08	−2·22	6·29 ± 0·06	−3·27
71°C SDPP at 30 s RT	6·73 ± 0·1	−3·29	5·55 ± 0·06	−4·01
71°C SDPP at 60 s RT	4·46 ± 0·14	−5·66	3·67 ± 0·13	−5·89
80°C SDPP at 0 s RT	7·61 ± 0·3	−2·41	5·45 ± 0·31	−4·11
80°C SDPP at 30 s RT	6·50 ± 0·14	−3·62	4·74 ± 0·02	−4·82
80°C SDPP at 60 s RT	4·82 ± 0·1	−5·3	4·21 ± 0·06	−5·35
Statistical analysis				
SEM	0·09		0·08	
Temp	0·51		<0·001	
Time	<0·001		<0·001	
Temp*time	0·009		<0·001	

SDPP, liquid porcine plasma spray‐dried at 200°C inlet temperature and either 71°C or 80°C outlet temperature; RT, residence time of postheating treatment after spray‐dry of 30 s (70·4°C) and 60 s (80·7°C); RF, Log10 reduction factor; SEM, standard error of the least square means; Temp, main effect of outlet temperature; Time, main effect of residence time; Temp*time, interaction of effects of Temp and Time.

**Figure 1:**
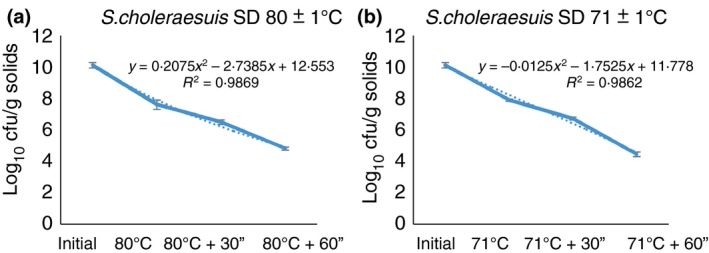
*Salmonella choleraesuis* viability in SDPP samples produced at an outlet temperature of 80 ± 1°C (a) or 71 ± 1°C (b) and held at different residence times. Dotted line provides the exact data obtained in the experiment. Solid line is the calculated linear regression from the data obtained. [Colour figure can be viewed at http://wileyonlinelibrary.com]

Plasma inoculated with *S. choleraesuis* and spray‐dried at inlet temperature of 200 ± 5°C and the two‐outlet temperatures indicated reduction in bacterial counts as shown in Table 1. A higher reduction of *S. choleraesuis* at 80°C outlet temperature was observed, although it was not statistically different (*P *=* *0·510) from 71°C outlet temperature. The effect of RT presented a log polynomic regression inactivation curve with an *r* ^2^ of 0·99 (Fig. 1a,b) for both outlet temperatures. Higher reduction (*P *<* *0·001) was observed with prolonged RT. In addition, storage of all dried samples at either 4·0 ± 3·0°C or room temperature (23·0 ± 0·3°C) for 15 days eliminated surviving *S. choleraesuis* in dried plasma regardless of the spray‐drying conditions or RT. When liquid plasma was inoculated with *S. choleraesuis,* stored in refrigerated temperature (4·0 ± 3·0°C), and seeded in TSA every 2 h for an 8‐h period postinoculum, the bacterial count was maintained at an average of 9·11 ± 0·05 cfu log_10_ ml^−1^ almost without variation during the entire 8‐h period.

**Figure 2:**
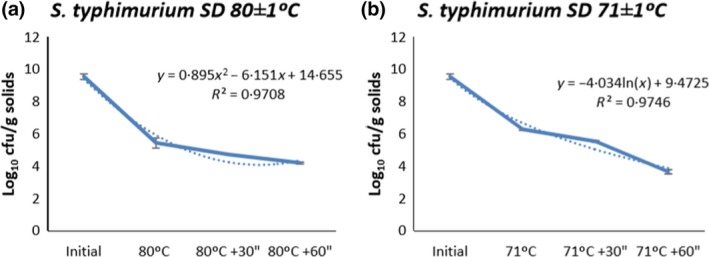
*Salmonella typhimurium* viability in SDPP samples produced at an outlet temperature of 80 ± 1°C (a) or 71 ± 1°C (b) and held at different residence times. Dotted line provides the exact data obtained in the experiment. Solid line is the calculated linear regression from the data obtained. [Colour figure can be viewed at http://wileyonlinelibrary.com]

*S. typhimurium* inoculated in plasma and spray‐dried at inlet temperature of 200 ± 5°C and outlet temperature of 80 ± 1°C or 71 ± 1°C had a reduction in bacterial counts as shown in Table 1. A significant higher (*P *<* *0·001) reduction of *S. typhimurium* for 80°C outlet temperature was found compared to 71°C outlet temperature. The inactivation kinetics presented a polynomic regression curve with an *r* ^2^ = 0·97 (Fig. 2a,b) for both outlet temperatures when the RT was applied. Also, a higher reduction of *S. typhimurium* with prolonged RT (*P *<* *0·001) was observed. When liquid plasma was inoculated with *S. typhimurium* and seeded in TSA plates, no significant changes in counts (8·69 ± 0·12 cfu log_10_ ml^−1^) over an 8‐h period were detected. Furthermore, as observed with *S. choleraesuis*, storage of all dried samples at either 4·0 ± 3·0°C or room temperature (23·0 ± 0·3°C) for 15 days eliminated surviving *S. typhimurium* in dried plasma independently of the spray‐drying conditions or RT.

This study showed that both spray‐drying conditions and extended RT postdrying had a significant effect on reducing the survival of both *Salmonella* spp. strains studied. In addition, storage time at either 4·0 ± 3·0°C or 23·0 ± 0·3°C for at least 15 days was shown to eliminate the remaining detectable viable bacteria.

During spray‐air contact, droplets interact with the hot air in the spraying chamber. Initially, as moisture is lost, the particle is maintained at the adiabatic wet‐bulb temperature, then, the droplet temperature increases to reach a value close or similar to the outlet air temperature (Straatsma *et al*. 2007; Perdana *et al*. 2013, 2015). Inlet and outlet temperature are the two main parameters that have a major influence on the inactivation of micro‐organisms. Inactivation occurs predominantly during the initial period of drying, while the remaining drying time further decreases the moisture content (Perdana *et al*. 2013, 2015). The survival of micro‐organisms is reduced by increasing the inlet temperature, but the outlet air temperature has the greatest impact on pathogen inactivation because this is the minimum temperature that the particle will achieve during the drying process; therefore, higher outlet temperature typically results in higher microbial inactivation (Perdana *et al*. 2013, 2015). Relatively high drying temperatures and rapid dehydration are two phenomena involved in microbial inactivation. Although the most important site of damage caused by dehydration is the cytoplasmic membrane (Crowe *et al*. 1987; Lievense and Van't Riet 1994), dehydration also produces damage to DNA/RNA and proteins (Lievense 1992). Results of the current study demonstrated that a greater reduction in both *Salmonella* spp. counts was observed at the higher outlet temperature, although it was only statistically significant for *S. typhimurium*. The minimum outlet spray‐drying temperature is 80°C for the commercial manufacturing process of SDP (Sampedro *et al*. 2015; Pérez‐Bosque *et al*. 2016) and results of the present study suggested that both *Salmonella* spp. strains were susceptible to spray‐drying even at a lower outlet temperature (71°C).

Laboratory spray‐dryers are useful for establishing guidelines to scale‐up the industrial production of SDP. The main differences between laboratory and pilot plant dryers compared with industrial dryers are design, size and volume processed, all of which affect the retention or dwell time of the product within the chamber (Foster and Leatherman 1995). Laboratory spray‐dryers have reduced retention or dwell time of the product within the chamber (<1 s) compared with commercial dryers (between 20 and 90 s, depending upon scale and design of the dryer). Furthermore, there is an immediate cooling to room temperature of the small quantity of dried product produced by lab dryers in comparison with industrial dryers which process much larger quantity of material that extends the time for dissipation of heat from the dried product. Present results indicated that when extended RT was simulated after drying liquid plasma with a lab dryer at temperatures around 71 or 80°C, there was a significant reduction in survival of both *Salmonella* spp. strains that was directly related with the higher RT regardless of outlet drying temperature. These results may confirm that commercial dryers may be more effective than lab dryers to inactivate micro‐organisms as suggested by Perdana *et al*. (2013).

Furthermore, SDBP are dry products with low moisture (<9%) and very low water activity (*a*_w_ < 0·6). Some pathogens, especially bacteria and enveloped viruses, are not able to survive for prolonged periods of time in dry materials like SDBP (Sampedro *et al*. 2015). Several mechanisms affecting microbial survival in dry materials have been described, such as, oxidative stress and reactive oxygen species formation which produces lipid peroxidation, and the browning reaction of sugars that cause protein denaturation and DNA damage. These changes are accumulative and have lethal effects on bacterial metabolism (Hernández García 2011). Therefore, as an additional safety feature, most manufacturers pack and store porcine SDBP at room temperature (>20°C) for at least 14 days before release for sale. These storage conditions have been demonstrated as effective to inactivate certain pathogens susceptible to dry environments and mild temperatures, such as PRRSV, PEDV and coronaviruses in general (Pujols and Segalés 2014; Sampedro *et al*. 2015). The present study showed that *S. choleraesuis* and *S. typhimurium* did not survive in dried samples of plasma stored for 15 days after production at 4·0 ± 3·0°C or 23·0 ± 0·3°C, regardless of the outlet temperature used during drying or the postdrying residence time.

Under the conditions of this study, the combinations of spray‐drying and RT followed by postdrying storage at 4·0 ± 3·0°C or 23·0 ± 0·3°C for 15 days were effective for eliminating detectable viable bacteria count of the two *Salmonella* spp. strains studied.

## Materials and methods

### Bacterial strains and test products

*Salmonella choleraesuis* (ref.: UMI‐UAB 46429) and *S. typhimurium* (ref.: UMI‐UAB 46450) strains were provided by the UMI‐UAB (Veterinary School, Infectious Diseases Unit, Universitat Autònoma de Barcelona, Spain). Inocula of both *Salmonella* spp. strains were prepared separately, growing one colony of each bacterium in TSA plates. After 24 h of growth at 37°C, bacteria were collected with a Kolle handle and resuspended in 10 ml PBS.

Commercial spray‐dried porcine plasma (SDPP; AP820P Lot # Y630962‐357, APC Europe S.L., Granollers, Spain) was sterilized by *γ*‐cobalt‐60 irradiation at 10 kGy (Aragogamma S.A., Les Franqueses del Vallés, Barcelona, Spain) to eliminate any potential bacterial contamination. The *γ*‐irradiated SDPP was diluted 1/10 in sterile distilled water (0·6 kg SDPP + 6 kg of water) to obtain 6·6 kg of liquid plasma containing around 8·5% solids. After solubilization, liquid plasma was passed through a sterile tissue to eliminate any insoluble material. Three 2 kg aliquots were obtained from the 6·6 kg of diluted plasma for spray‐drying. A 10‐ml inoculum of each bacterium containing around 10^10^–10^12^ CFU ml^−1^ was prepared and used to inoculate each 2·0 kg aliquot of plasma to achieve a minimum final titre of approximately 10^8^–10^10^ CFU ml^−1^. This procedure was conducted in triplicate for each bacterium and was done in a sterile biological safety cabinet to avoid external contamination.

### Spray‐drying test

Two kilograms of resuspended SDPP (8·60 ± 0·01% solids) were inoculated with 10 ml of either the *S. choleraesuis* or the *S. typhimurium* isolates. From each aliquot of 2 kg of inoculated plasma, two bottles of 1 kg were obtained and one bottle was spray‐dried at an inlet temperature of 200 ± 5°C and an outlet temperature of 80 ± 1°C and the other 1 kg bottle was spray‐dried at the same inlet temperature with an outlet temperature of 71 ± 1°C. Before drying the inoculated plasma, the spray‐dryer was stabilized with water followed by non‐inoculated plasma to achieve the combination of inlet and outlet temperatures of interest (Büchi Mini Spray Dryer B‐290, Büchi Labortechnik, Switzerland). All inlet and outlet temperature combinations were performed in triplicate. Air flow through the column was set at 20‐27 m^3^ h^−1^ at 20°C. Estimated dwell time was <1 s. Before spray‐drying, each inoculated bottle was sampled for bacterial plate count and solids analysis.

Once SDPP was obtained at the two designated outlet temperatures, each dried spiked sample was distributed in 27 glass tubes (0·5 cm length; inner diameter of 8 mm) containing 0·5 g of product. Three tubes were immediately seeded on TSA plates for bacterial count. Three samples were immediately stored at room temperature (23·0 ± 0·3°C) and 3 more samples were immediately stored at refrigerated temperature (4·0 ± 3·0°C) for 15 days, and then analysed for bacterial count.

**Figure 3:**
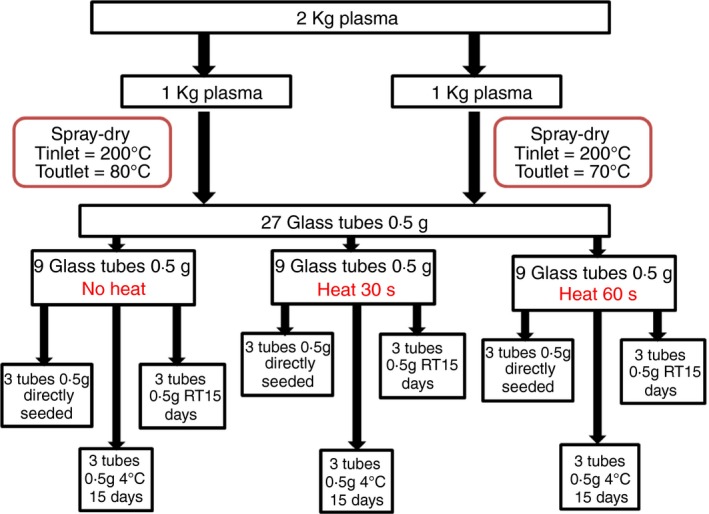
Experimental design of the study. One kilogram of plasma was spray‐dried at an outlet temperature of 80 ± 1°C and another kg was spray‐dried at an outlet temperature of 71 ± 1°C. The same procedure was conducted in triplicate. In addition, each tube was analysed in triplicate. RT, Room Temperature. [Colour figure can be viewed at http://wileyonlinelibrary.com]

Particle residence time (RT) in a laboratory spray‐dryer is typically <1 s and particles cool very rapidly, whereas commercial dryers have a RT ranging from 30–60 s thus particles have an extended time of heat exposure. To simulate the longer RT typical of commercial dryers, 9 dried samples kept in sealed glass tubes were placed in a water bath set at 89–91°C for a RT of 30 s (actual temperature of the powder sample was 70·4°C) and 9 more samples were held for a RT of 60 s (actual temperature of the powder sample was 80·7°C). Upon completion of each RT, three tubes of samples held at either 30 or 60 s RT were stored at room (23·0 ± 0·3°C) or refrigerated (4·0 ± 3·0°C) temperature for 15 days before being analysed for bacterial count. The study design is summarized in Fig. 3. These procedures were conducted in triplicate for each *Salmonella* strain.

Bacterial count was done on TSA plates in triplicate for both liquid and dried samples. Each tube containing 0·5 g of dried sample was resuspended in sterile water at 1:9 ratio. From this resuspension, 0·1 ml was seeded in TSA agar for 24 h at 37°C. The colony counts were done following the ISO 7218:2007 guidelines. Results were expressed as a log_10_ g^−1^ of solids using the equation: log_10_ g^−1^ = log_10_ (CFU/ml/[(% solid content of resuspended sample)/100].

Liquid inoculated plasma samples were analysed immediately after inoculation but also during an 8 h‐period after inoculation to determine if the liquid plasma had an effect on reducing *Salmonella* spp. survival independently of the spray‐drying effect. The liquid samples were maintained at 4·0 ± 3·0°C during this period and analysed for every 2 h.

Liquid and spray‐dried samples were analysed for dry matter (AOAC method 925·45) to allow expression of the microbial inactivation results by grams of solids.

### Statistical analysis

Data were expressed by means of Log_10_ values and standard deviations of three independent experimental batches. Experimental data were analysed as a 2 × 3 factorial arrangement of treatments using PROC GLM of SAS (SAS Institute. Cary, NC). Independent factors were outlet temperature (80 *vs* 71°C) and residence time (0, 30 or 60 s). Least square means were reported and differences at *P *<* *0·05 were considered significant.

## Conflict of Interest

Elena Blázquez, Carmen Rodríguez, Jesús Ródenas, Marc Solà‐Ginés and Javier Polo are employed by APC Europe, S.L.U. Joy Campbell and Javier Polo are employed by APC Inc. Both companies manufacture and sell spray‐dried animal plasma. Joan Pujols, Ana Pérez de Rozas and Joaquim Segalés declare no conflicts of interest.
